# Diversity and Biogeography of Bathyal and Abyssal Seafloor Bacteria and Archaea Along a Mediterranean—Atlantic Gradient

**DOI:** 10.3389/fmicb.2021.702016

**Published:** 2021-11-01

**Authors:** Blandine Trouche, Miriam I. Brandt, Caroline Belser, Covadonga Orejas, Stéphane Pesant, Julie Poulain, Patrick Wincker, Jean-Christophe Auguet, Sophie Arnaud-Haond, Loïs Maignien

**Affiliations:** ^1^Univ Brest, CNRS, IFREMER, Microbiology of Extreme Environments Laboratory (LM2E), Plouzané, France; ^2^MARBEC, Univ Montpellier, Ifremer, IRD, CNRS, Sète, France; ^3^Génomique Métabolique, Genoscope, Institut François Jacob, CEA, CNRS, Univ. Évry, Université Paris-Saclay, Evry, France; ^4^Centro Oceanográfico de Baleares, Instituto Español de Oceanografía, Palma de Mallorca, Spain; ^5^European Molecular Biology Laboratory, European Bioinformatics Institute, Cambridge, United Kingdom; ^6^MARBEC, Univ Montpellier, CNRS, IFREMER, IRD, Montpellier, France; ^7^Marine Biological Laboratory, Josephine Bay Paul Center for Comparative Molecular Biology and Evolution, Woods Hole, MA, United States

**Keywords:** biogeography, distance-decay relationship, dispersal limitation, drift, benthic microbiology, seafloor sediment, bathyal zone, metabarcoding

## Abstract

Seafloor sediments cover the majority of planet Earth and microorganisms inhabiting these environments play a central role in marine biogeochemical cycles. Yet, description of the biogeography and distribution of sedimentary microbial life is still too sparse to evaluate the relative contribution of processes driving this distribution, such as the levels of drift, connectivity, and specialization. To address this question, we analyzed 210 archaeal and bacterial metabarcoding libraries from a standardized and horizon-resolved collection of sediment samples from 18 stations along a longitudinal gradient from the eastern Mediterranean to the western Atlantic. Overall, we found that biogeographic patterns depended on the scale considered: while at local scale the selective influence of contemporary environmental conditions appeared strongest, the heritage of historic processes through dispersal limitation and drift became more apparent at regional scale, and ended up superseding contemporary influences at inter-regional scale. When looking at environmental factors, the structure of microbial communities was correlated primarily with water depth, with a clear transition between 800 and 1,200 meters below sea level. Oceanic basin, water temperature, and sediment depth were other important explanatory parameters of community structure. Finally, we propose increasing dispersal limitation and ecological drift with sediment depth as a probable factor for the enhanced divergence of deeper horizons communities.

## Introduction

Marine sediments cover around 65% of the Earth’s surface and accumulate particulate organic matter settling from the water column, thereby representing the largest sink of oceanic organic matter (Seiter et al., 2005; Jørgensen and Boetius, 2007). Bacteria and archaea in these sediments represent the largest pool of biomass in the deep sea, with their abundance estimated to be on the order of 4.9 × 10^28^ cells in the benthic layer (top 50 cm) and 2.9 × 10^29^ globally (Kallmeyer et al., 2012; Danovaro et al., 2015). Contrary to meio-, macro-, and mega-fauna, their abundance and biomass does not decrease with water depth, though cell counts decrease logarithmically with depth in the sediments. Benthic bacteria and archaea are essential for the early diagenesis of sinking organic matter and as a consequence, they are crucial contributors to biogeochemical cycles, determining the partitioning between buried organic matter and nutrients released in the water column (Orcutt et al., 2011; Teske et al., 2011). This underlines the importance of the benthic boundary layer microbial communities as a transition between water-column and subseafloor communities (Zinger et al., 2011; Walsh et al., 2016).

Thanks to recent technological advances, particularly in sequencing techniques (e.g., Huber et al., 2006, reviewed in Salazar and Sunagawa, 2017), it is now possible to perform near-exhaustive inventories of benthic microbial community diversity across large spatial scales, and to investigate patterns of microbial distribution. Despite their essential role in the marine ecosystem (Nealson, 1997; del Giorgio and Duarte, 2002; Jørgensen and Boetius, 2007; Arístegui et al., 2009; Molari et al., 2013), processes shaping benthic prokaryotic community structure are still poorly understood, and the existence of biogeographic patterns has been questioned owing to their possible unlimited dispersal ability (Green and Bohannan, 2006; Astorga et al., 2012). Nonetheless, recent studies focusing on deep sea benthic microorganisms at local and regional scale (Jacob et al., 2013; Buttigieg and Ramette, 2015; Liu et al., 2020; Li et al., 2021) and meta-analyses (Bienhold et al., 2016; Petro et al., 2017; Hoshino et al., 2020) have clearly shown geographic structuration in these communities, even at reduced spatial scales.

Biogeographic patterns are usually considered to result from four main evolutionary forces: selection, diversification, dispersal, and drift (Vellend, 2010; Hanson et al., 2012; Nemergut et al., 2013). These processes are often split between deterministic and stochastic, selection being considered wholly deterministic, drift being stochastic, and dispersal and diversification largely accepted as stochastic processes, although they may encompass both deterministic and stochastic components (Zhou and Ning, 2017). One of the most studied biogeographic patterns resulting from these processes is the evolution of community composition with geographic distance. When community similarity decreases with increasing geographic distance, a distance-decay relationship (DDR) or “isolation by distance” pattern will be observed (Nekola and White, 1999; Horner-Devine et al., 2004; Soininen et al., 2007; Hanson et al., 2012). Coupled with investigation of the link between community and environmental similarity, this approach provides insights into the relative contribution of historical and contemporary processes shaping microbial provinces and habitats, as proposed by Martiny et al. (2006).

Besides, microbial communities display a strong stratification with sediment depth that has traditionally been explained by the redox gradient with depth of electron acceptors that are sequentially consumed by organic matter respiring microorganisms (Emerson et al., 1980; Durbin and Teske, 2011; Orcutt et al., 2011). In addition to the deterministic influence of environmental conditions, recent studies focusing on processes involved in vertical distribution of sedimentary microorganisms have suggested a strong influence of surface community structure on the subseafloor community assembly through selective survival, beginning in the very first layers of sediment (Jochum et al., 2017; Petro et al., 2017, 2019; Starnawski et al., 2017; Kirkpatrick et al., 2019; Marshall et al., 2019).

In this study, we aimed at examining benthic microbial community diversity and biogeographic patterns across the Mediterranean - Atlantic basins to determine to what extent the microbial community structure resulted from past historical processes vs. contemporary environmental drivers at different spatial scales. Building on previous work suggesting that assembly of subseafloor microbial communities initiates in the very first layers of sediment, we also examined the evolution of microbial community structure with increasing depth in the surface sediments of the seafloor.

## Materials and Methods

### Sample Collection and Processing

#### Cruises and Locations

**FIGURE 1:**
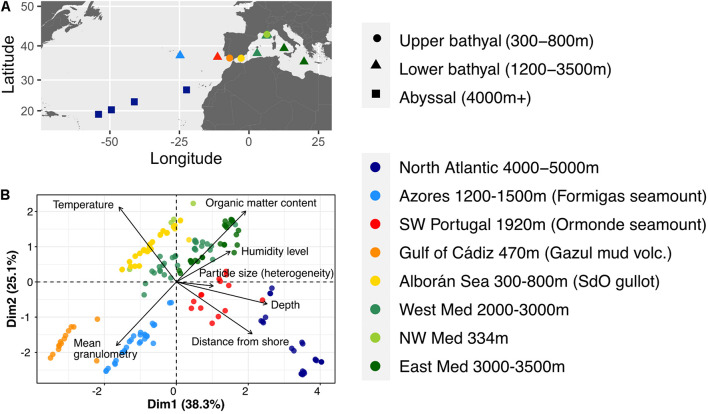
**(A)** Map of the sampling stations across the Mediterranean and Atlantic transition. **(B)** Characterization of the samples based solely on available metadata using a principal components analysis biplot. Arrows represent the decomposition of the variables along the 2 first dimensions, with their length illustrating the cos^2^ or quality of representation of the variables with these dimensions.

Samples from 18 stations from the eastern Mediterranean Sea to the northern Atlantic Ocean were collected between April 2016 and May 2017 (Figure 1A). In the spring of 2016, samples were taken from the upper and lower bathyal zones of the Gulf of Lion during cruises ESSNAUT16 (DOI: 10.17600/16000500) and CanHROV (DOI: 10.17600/16012300). In September 2016, the MEDWAVES cruise (Atlas project H2020) targeted one Mediterranean feature (Seco de los Olivos gullot), and three Atlantic features (Gazul mud volcano in the Gulf of Cádiz, Ormonde seamount off Portugal and Formigas seamount off Azores) (Orejas et al., 2017). In March 2017, samples were collected from the abyssal plains of the North Atlantic Ocean during transect cruise AMIGO1. Finally, in May 2017, the sampling for this study was completed during the PEACETIME cruise (DOI: 10.17600/17000300), targeting the lower bathyal zone of the western Mediterranean Sea. Details of the stations are given in Table 1.

**TABLE 1:** List of sampling stations and their characteristics.

Geographic zone	Location	Latitude	Longitude	Depth (mbsl)	Station name	Sampling cruise
Mediterranean Sea	Mediterranean (abyssal plain)—Ionian Sea	35.4891	10.776	3,100	ION	PEACETIME
	Mediterranean (abyssal plain)—Tyrrhenian Sea	39.3402	12.5927	3,400	TYRR	
	Mediterranean (undersea canyon)	42.7167	6.1333	2,490	Canhrov_ST1	CanHROV
	Mediterranean (passive margin)—Gulf of Lion	42.9422	6.7422	2,417	ESN1	ESSNAUT16
		43.0867	6.4512	334	ESN2	
	Mediterranean (abyssal plain)	37.9467	2.9167	2,800	FAST	PEACETIME

Transition	Alborán Sea (Seco de los Olivos gullot)	36.4808	–2.8945	729	ST179	MEDWAVES
		36.5460	–2.8135	381	ST201	
		36.5157	–2.7942	554	ST215	
	Gulf of Cádiz (Gazul mud volcano)	36.5598	–6.9492	470	ST22	
		36.5605	–6.9498	470	ST23	
	Southwest Portugal (Ormonde seamount)	36.8442	–11.3025	1,920	ST38	

Atlantic Ocean	Azores (Formigas seamount)	37.34	–24.7552	1,325	ST117	
		37.2837	–24.7873	1,245	ST68	
	North Atlantic (abyssal plain)	26.89	–22.4442	4,931	Amigo1_ST0	AMIGO1
		23.0042	–41.2092	4,770	Amigo1_ST1	
		20.3392	–49.4359	4,630	Amigo1_ST2	
		18.8175	–54.0836	4,630	Amigo1_ST3	

*Mbsl, meters below sea-level.*

#### Sampling Protocol

For each station, three cores were collected with a multicorer (MUC) or push-cores deployed from the Nautile submarine (ESSNAUT16) or a remotely operated vehicle (ROV, CanHROV). The sediment cores were sliced onboard in a lab environment previously cleaned using ∼10% bleach solution, rinsed with ethanol and ultrapure water. Each core was sliced into depth layers following a standard scheme: 0–1, 1–3, 3–5, 5–10, and 10–15 cm, and when the cores were long enough 15–30 cm or to 1 cm before the maximum length, to avoid contamination from the core extruder. Slicing was performed using spatulas also bleached and rinsed with ultrapure water before each use. Horizons (slices of sediment) were transferred into zip-lock bags, homogenized, and frozen at −80°C on board before being shipped on dry ice to the laboratory where they were also kept at −80°C.

### DNA Extraction

DNA extractions were performed in a sterile lab, using approximately 10 g of sediment with the PowerMax Soil DNA Isolation Kit according to the manufacturer’s instructions (Qiagen, Hilden, Germany) with modifications: the elution buffer was left on the spin filter membrane for 10 min at room temperature before centrifugation in order to increase DNA yield. Extraction controls were performed by using an empty tube from the kit for each series of extraction or extraction kit batch. In total, 8 extraction blanks were produced. When field controls were prepared onboard (empty zip-lock bags), the first solution of the kit was poured into the control ziplock bag, before following the usual extraction steps. Each of the resulting 5 mL DNA solutions were stored at −80°C.

### Libraries Construction and Sequencing

A primer pair targeting both Bacteria and Archaea (Parada et al., 2016) was used to amplify the V4V5 region of the 16S rRNA gene (515F: 5′-GTGYCAGCMGCCGCGGTAA, 926R: 5′-CCGYCAATTYMTTTRAGTTT). PCR amplifications were carried out at Genoscope (Evry, France) as part of the eDNAbyss project (see Supporting Information for amplification, purification, and quantification details). Amplicon libraries were prepared for each sample by non-directional ligation of Illumina adapters on 100 ng of amplicons following the Kapa Hifi HotStart NGS library Amplification kit (Kapa Biosystems, Wilmington, MA, United States). After quantification and quality control, libraries normalized to 8–9 pM concentrations and containing a 20% PhiX spike-in were sequenced on HiSeq2500 instruments in a 250 bp paired-end mode (System User Guide Part # 15035786).

### Bioinformatic Analysis

All bioinformatic analyses were performed using a standardized pipeline (Brandt et al., 2021), available on Gitlab,^1^ on a home-based cluster (DATARMOR, Ifremer).

First, sequence files were renamed from their Genoscope identifiers to more explicit names. Due to non-directional adapter ligation, inserts were sequenced in different orientations. We thus used Cutadapt v1.9 (Martin, 2011) to identify the primer sequence in each read and sort them according to two criteria: forward or reverse primer and forward or reverse sequencing. Data for each sample was thus split into 4 sequence files (R1F, R1R, R2F, R2R). Cutadapt then removed the identified primer sequences and BBMAP repair (Bushnell, 2014) was used to ensure that reads were still paired by sorting reads using the information present in their description line and removing unmatched reads.

For each sequencing run, we determined Amplicon Sequence Variants, merged read pairs and removed chimeras using the DADA2 package v.1.10 (Callahan et al., 2016), following guidelines from the online tutorial for paired-end HiSeq data.^2^ The script implementing DADA2 was applied separately to the two pairs of sequence files R1F/R2R and R2F/R1R. The parameters used for filtering and trimming reads were as follows: truncation length of 220 base pairs, maxN = 0, maxEE = 2 and truncQ = 11. The error learning step was based on nbases = 1e8. Merged sequences were size-filtered by keeping sequences with a length between 350 and 390 bps.

The Amplicon Sequence Variants (ASVs) tables produced by DADA2 for each run were then merged, collapsing ASVs based on DNA sequence identity. Taxonomic assignment was performed with the implementation of the RDP naive Bayesian classifier (Wang et al., 2007) available in DADA2 v.1.10, using the Silva v138 reference database (Quast et al., 2013) and a bootstrap threshold of 80.

The ASV and taxonomy tables produced by this pipeline were then combined in a phyloseq object (phyloseq v1.28.0, McMurdie and Holmes, 2013) in an R v3.6.1 environment. Reads from the same amplicon library, but originating from different Illumina runs, were merged under the same sample name before removing sequences from unwanted taxa (Eukaryota, Chloroplast and Mitochondria affiliated sequences). Data was decontaminated using extraction, PCR and field controls using the decontam package (v1.4.0, Davis et al., 2018), or handpicking in the case of the ASV dominating control libraries reads (see reproducible workflow on github). Samples totaling less than 40,000 reads after decontamination were removed, the appropriate metadata added (Supplementary Table 1), and the final object saved as a phyloseq object for further analysis in R.

Scripts for the reproducible bioinformatic workflow are available at https://loimai.github.io/ABYSS_16S/.

### Sediments Characterization

Characterization of the sediment samples was carried out by Filab S.A.S (Dijon, France). Granulometry values were obtained using wet Malvern laser scattering together with humidity level and loss on ignition at 550°C (see Supporting Information for more details on the methods used).

Temperature for each sampling station was extrapolated when possible (MEDWAVES expedition) from CTD data from the same sampling stations (Orejas et al., 2017). When this data did not exist, it was set to average temperature recorded in the ocean basin at the depth considered (Mantyla and Reid, 1983; Pierre, 1999; Martín-Cuadrado et al., 2007).

### Statistical Analysis

All subsequent statistical analyses were done in R v3.6.1, using phyloseq (v1.28.0, McMurdie and Holmes, 2013), vegan (v2.5.7, Oksanen et al., 2020), and ggplot2 (v3.3.0, Wickham, 2016) packages to compute alpha diversity, beta diversity, and produce taxonomy barplots.

A map of the sampling stations was generated using ggplot2 (v3.3.0, Wickham, 2016). Description of the sampling sites was based on available metadata using the principal components analysis (PCA) from package FactomineR (Lê et al., 2008). Environmental parameters considered for each sample were ocean depth at sampling station, distance from shore, sediment horizon (depth in the sediment core), temperature above seafloor, and sediment characteristics, namely mean organic matter content by station and horizon, mean humidity level by station and horizon, mean granulometry (μm), and heterogeneity of particle size. Visualization of the DDRs relied on community similarity computed using a Bray-Curtis index after normalization of the dataset using cumulative sum scaling with the metagenomeSeq package (Paulson et al., 2013). Geographic distance was measured using a “Vincenty” (ellipsoid) great circle distance to take into account Earth curvature, relying on packages enmSdm, and geosphere (v1.5.10, Hijmans, 2019). Environmental similarity between samples was estimated with euclidean distances on the centered and scaled environmental data table, using the R base *scale* function. The statistical difference between the slopes of the models fitted to the DDRs was tested using function *diffslope2* from package simba (Jurasinsk and Retzer, 2012).

Beta-diversity variation partitioning analysis were computed using function *varpart* in the vegan package. Ordinations of the microbial data were visualized as nMDS using phyloseq, and environmental data were fitted to the ordinations using the *envfit* function in package vegan. Permutational multivariate analyses of variance were performed when appropriate with the *adonis* function of package vegan, after checking the homogeneity of group dispersions using function *betadisper*. Finally, biomarker detection was done with the DESeq2 package (1.24.0, Love et al., 2014). The fully reproducible workflow for statistical analysis is available at: https://loimai.github.io/ABYSS_16S/.

## Results

### 16S rRNA Gene Amplicon Processing

A total of 230 sample libraries were built and sequenced, producing 195,470,177 raw sequences of the 16S rRNA gene’s V4-V5 region, with a mean of 849,870 reads by library. A total of 17,366,268 reads were recovered from the additional 24 control libraries that were constructed and sequenced simultaneously, originating from sampling (empty storage bags conditioned on-board research vessels at the end of some of the sampling sessions), extraction (empty kit processed through all extraction steps together with the samples) and PCR (ultrapure water) blanks (Supplementary Table 1).

After processing with DADA2 the dataset included 123,454,421 sequences for a total of 265,198 ASVs. Of those, 1,223 (about 0.5%) were found in control samples, and 728 ASVs were specific to the control libraries. Most of the contamination was dominated by a specific ASV that accounted for 99% of reads in negative control libraries. This ASV, affiliated with partial 16S sequences of Sphingobium strains, is a recognized contaminant of Taq-Phusion reagents (Salter et al., 2014).

After bioinformatic processing, taxonomic refining and decontamination, the dataset comprised a total of 210 libraries including 66,826,975 sequences representing 260,567 ASVs (min 40,076 sequences, max 847,227 sequences) (Supplementary Table 2). Rarefaction curves (Supplementary Figure 1) confirmed that sequencing and sampling efforts captured most of the sample diversity.

### Description of Sampling Sites

Three geographic zones were defined based on the coordinates of the sampling stations (Table 1 and Figure 1A). From East to West, the Mediterranean zone grouped the stations from the Ionian and Tyrrhenian Sea, the Gulf of Lion and the abyssal plain near the Balearic Islands. The Transition zone around the Gibraltar Strait consisted of the stations from the Alborán Sea, Gulf of Cádiz and southwest Portugal. Finally, the Atlantic zone was composed of the Azores and north Atlantic abyssal plain stations.

We first characterized the samples based on the available environmental parameters (depth, distance from shore, temperature, organic matter (OM) content, humidity level, mean granulometry (μm) and heterogeneity of particle sizes) (Figure 1B). The first two dimensions of the PCA summed up 63.4% of the total inertia. Five variables contributed most to these dimensions, namely depth and distance from shore, temperature, OM content and granulometry, leading to a segregation of samples by site rather than oceanic basin. Depth and distance from shore were anti-correlated with temperature, thus creating a gradient of sampling sites from the shallow warm sediments of the Mediterranean Sea to the deep abyssal samples of the Atlantic Ocean. Two groups of sites, from the Azores and Gulf of Cádiz, differed most from the others based on the sediment composition data.

### Distance-Decay Relationship Between Deep Sea Sediment Communities

**FIGURE 2:**
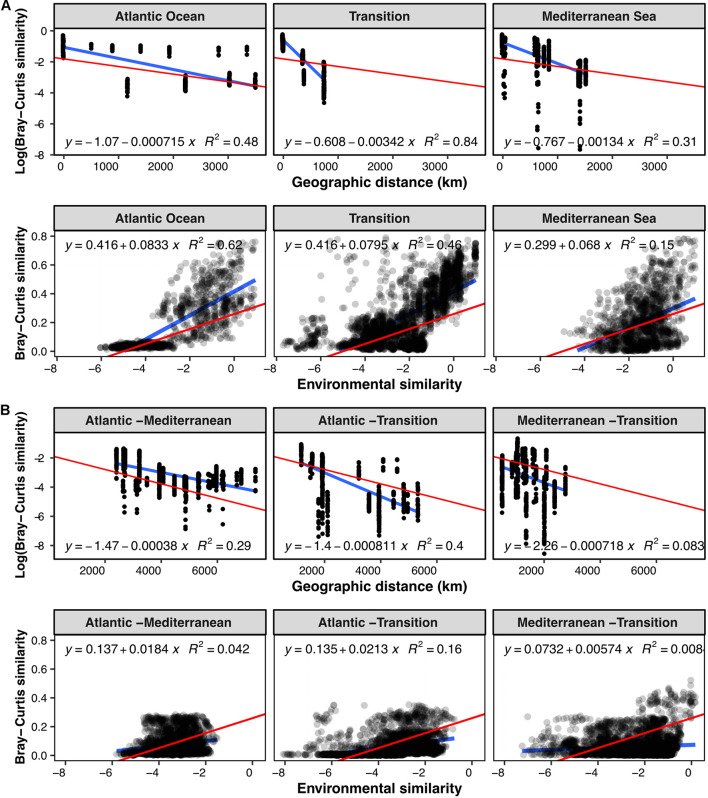
Pairwise Bray-Curtis community similarity between samples with respect to geographic distance (km) and environmental similarity: **(A)** samples from the same geographic zone, **(B)** samples from two different zones. For the evolution with distance, pairwise community similarity was evaluated exclusively between samples of the same horizon. Blue lines illustrate linear models computed for the subset of samples considered, and red lines represent the overall linear regression when including all the samples. All linear models have a *p*-value at least inferior to 3.306e^–10^. Overall linear regression Log (Bray-Curtis) vs. geographic distance: *y* = –1.81–0.000491x, *R*^2^ = 0.27, *p* < 2.2e^–16^. Overall linear regression Bray-Curtis vs. environmental similarity: *y* = 0.261 + 0.0524x, *R*^2^ = 0.27, *p* < 2.2e^–16^.

To explore biogeographic patterns along the longitudinal gradient, we plotted the community similarity between pairs of samples as a function of their geographic distance and their environmental similarity (Figure 2). Regarding geographic distances, we only compared samples originating from the same sediment layer (horizon) and partitioned the pairwise comparisons according to sampling region (Mediterranean, Atlantic and Transition region) to investigate biogeographic patterns at regional (Figure 2A) and inter-regional scales (Figure 2B).

Community similarity between sample pairs generally decreased with geographic distance, hence exhibiting a clear DDR at all scales, both within (Figure 2A) and between geographic zones (Figure 2B). Linear regressions of the DDRs showed that the highest rate of decrease in community similarity with distance occurred in the transition zone (slope: –0.00342), followed by the Mediterranean basin (slope: –0.00134), and the Atlantic basin (slope: –0.000715). At inter-regional scale, the slope of the DDR was steepest between the Atlantic and Transition regions and the Mediterranean and Transition regions (Figure 2B). It was 0.5 times less steep in the Atlantic-Mediterranean comparison, likely due to the most distant samples originating from the deepest stations in the dataset (water depth).

In addition, we observed a generally positive correlation between community similarity and environmental similarity, with a modeled regression slope at least four times higher at intra-regional scale (Figure 2A, slope: 0.0703–0.0835) than at inter-regional scale (Figure 2B, slope: 0.00601–0.0213).

**FIGURE 3:**
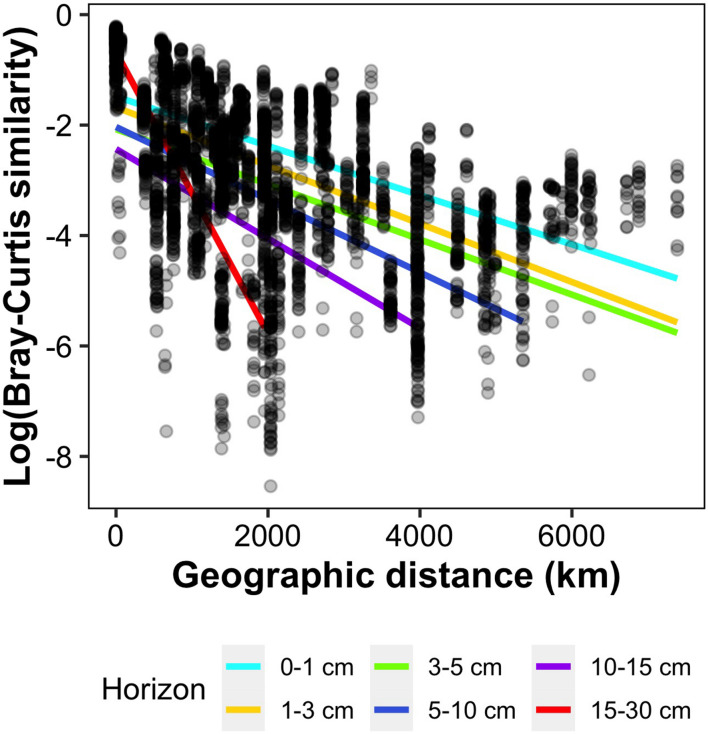
Pairwise Bray-Curtis community similarity with respect to geographic distance (km) between samples. Each point corresponds to a pairwise comparison between samples of the same sediment horizon, and a linear regression has been computed separately for each horizon (equations in Supplementary Table 3).

When decomposed for each sediment layer, we observed a clear increase in DDR with sediment horizon, with linear regression slopes approximately 5 times steeper in horizon 15–30 cm compared to the top three horizons (Figure 3 and Supplementary Table 3). Indeed, slopes ranged from 0.000445 in the first horizon (0–1 cm) to 0.00255 in horizon 15–30 cm (Supplementary Table 3), and were significantly different between adjacent horizons, except for horizons 1–3 and 3–5 cm. The fit of the regression did not clearly improve in deeper horizons, and ranged from 0.17 to 0.4, except for the lowest horizon where it reached 0.97 but was calculated on the lowest number of samples (*n* = 12).

### Environmental Parameters Structuring Microbial Communities

In terms of alpha-diversity, Shannon indices were comparable across locations, except in the north Atlantic and eastern Mediterranean abyssal plains (Supplementary Figure 2B), where Shannon diversity was significantly lower (Kruskal-Wallis rank sum test, *p* < 2.2e^–16^). These seemingly low diversity samples were the ones most affected by the Taq-Phusion contamination, most probably due to the low DNA content in the sediment and in the extract, resulting in poor sequencing depths. Alpha-diversity estimates decreased with increasing horizon depth for all locations, except for the sites located above 800 m ocean depth (Alborán Sea, Gulf of Cádiz, and NW Med) (Supplementary Figure 2B).

Overall, benthic microbial communities considered in this study exhibited similar dominant phyla, regardless of the origin of the samples in terms of geography, water depth, or horizon depth. Members of the Acidobacteria, Crenarchaeota (mostly Nitrososphaeria, previously a member of phylum Thaumarchaeota), Planctomycetota, and Proteobacteria (shared between Alpha- and Gammaproteobacteria) were predominant in all samples (Supplementary Figure 2A).

**FIGURE 4:**
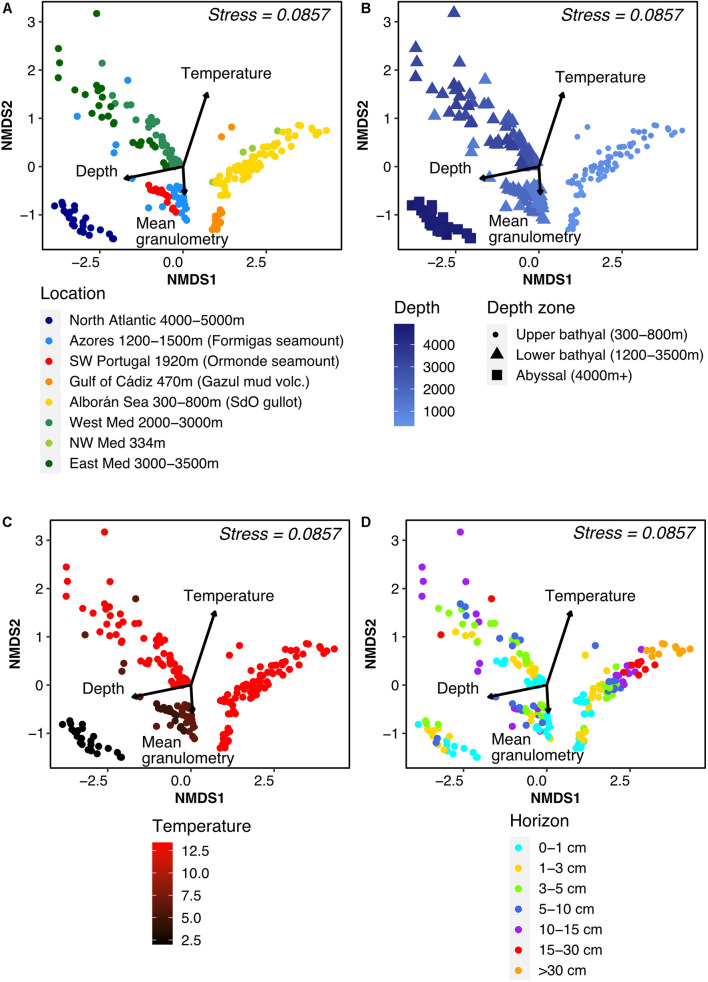
Non-Metric Multidimensional Scaling ordination plot of Bray-Curtis distance between samples, colored according to **(A)** geographic region, **(B)** water depth at sampling station (mbsl), **(C)** temperature of aboveground water (°C) and **(D)** horizon depth in the sediment core (cm). Environmental variables were fitted to the unconstrained ordination and the significant ones were added to the plot with their length illustrating the strength of the correlation.

When investigating the correlation between microbial community composition and environmental parameters, we found that samples readily clustered by site on the ordination (Figure 4A, PERMANOVA test *F* = 10.772, *p* = 0.001), as expected from the PCA results (Figure 1B). We also noticed a clear split between samples originating from shallow [<800 meters below sea-level (mbsl)] and deep (>1,200 mbsl) stations even when originating from the same oceanic basin (i.e., the Mediterranean). This depth effect was visible on ordination plots (Figures 4A,B), with a significant fit of the environmental data (*p* = 0.005), and on alpha diversity profiles (Supplementary Figure 2B). This difference was backed up by a variation partitioning analysis (Supplementary Figure 4). Focusing on samples deeper than 1,200 mbsl, the gradual change in community structure with water depth was maintained, with an increased turnover with changes in temperature and oceanic basin (Figures 4A–C).

Few differences in taxonomic composition were observed at phylum level between samples above and below 1,000 mbsl (Supplementary Figure 2A), except for an increasing importance of Acidobacteria with water depth, while Bathyarchaeia and Desulfobacterota (previously Deltaproteobacteria) were present almost exclusively above 1,000 mbsl.

Stratification of community composition with sediment horizon was observed in each depth range (Figure 4D) and was clearest in terms of taxonomy for the upper bathyal zone, with the following trends: increase in the presence of Acidobacteria, Chloroflexi, and Nanoarchaeota with increasing depth in the sediment cores, while the relative abundance of Alpha- and Gammaproteobacteria decreased (Supplementary Figure 2A). This horizon effect was mostly apparent inside each site (PERMANOVA *F* = 5.11, *p* = 0.001).

Contrary to the results from the environmental PCA (Figure 1B), sediment composition was not strongly linked with community composition. Indeed, humidity and heterogeneity of particle size were not significantly correlated with the ordination axes. Granulometry, and the anti-correlated OM content, were significantly (*p* = 0.01) correlated with axis 2 of the ordination though more weakly than temperature or depth. These results were reflected in the variation partitioning analyses (Supplementary Figure 4C).

### Exploring the Link Between Surface and Subsurface Communities at Local Scale

We chose a subset of three sites located in the Alborán Sea (Seco de los Olivos gullot), each sampled with triplicate cores down to 45 cm, to examine community changes at local scale. The sites were sampled at three water depths (381, 554m, and 729 m, see Table 1), with a maximum distance of 11 km between two sites.

**FIGURE 5:**
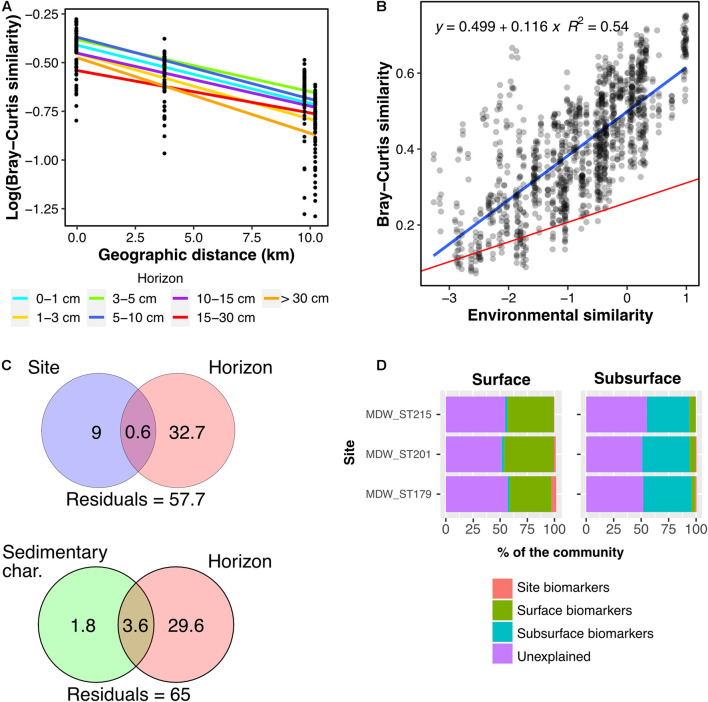
Pairwise community similarity between Alborán Sea samples as a function of **(A)** geographic distance (km), modeled by horizon, and **(B)** environmental similarity. In plot **(B)**, the blue line illustrates the linear regression for the Alborán Sea samples (*p* < 2.2e^– 16^), and the red line the regression for the complete dataset (*p* < 2.2e^– 16^). **(C)** Variation partitioning analysis for the Alborán Sea samples. **(D)** Relative composition of communities of the three sites of the Alborán Sea (Western Mediterranean Sea) based on the five sets of biomarkers identified: overall surface and subsurface biomarkers, and site-specific biomarkers (considering only surface horizons). Horizons between 0 and 10 cmbsf were considered surface horizons, and horizons deeper than 10 cmbsf were considered as subsurface horizons.

We observed a DDR for Alborán Sea samples (Figure 5A). However, in contrast with (inter)regional scales, neither the slope of the DDR nor its fit increased with depth in the sediment at local scale (Supplementary Table 4). A significant correlation between Bray-Curtis and environmental similarities was apparent as well, with a fit (*R*^2^ = 0.53) (Figure 5B) in the same range as what was observed at the regional scale (*R*^2^ = 0.16–0.62) (Figure 2A).

Variation partitioning analysis showed that a larger fraction of variation in data was attributed to the horizon effect (29.6–32.7%) than to site effect, conflating spatial distance and variation in depth and temperature (9%), or sediment composition (1.8%) (Figure 5C).

Biomarker analysis strengthened this observation (Figure 5D). We split our samples between surface horizons [0–10 centimeters below the seafloor (cmbsf)] and subsurface horizons (10–40 cmbsf), based on the findings of Petro et al. (2019) highlighting a shift in community composition at the bottom of the bioturbation zone. We then determined three sets of site-specific surface biomarker ASVs, together with one set of general surface biomarkers, and one set of general subsurface biomarkers. Represented in Figure 5D is the contribution of each set of biomarkers to the communities at each site, in the surface (left) and subsurface (right) horizons. Site-specific biomarkers made up a small fraction of the community (<1.8%) while surface and subsurface ones accounted for 36.9–45.6%. As seen in the taxonomic profiles, the surface biomarkers were assigned to a variety of taxonomic groups, including classes Nitrososphaeria, Alpha- and Gamma-proteobacteria, while the subsurface biomarker ASVs mostly belonged to phyla Desulfobacterota, Acidobacteria, Chloroflexi, and class Bathyarchaeia (Supplementary Figure 3A).

## Discussion

In this work, using a rigorous, standardized slicing scheme and homogeneous molecular and bioinformatic analyses, we confirm at local, regional, and inter-regional scales the existence of strong biogeographic patterns for prokaryotic communities across the Mediterranean-Atlantic transition. Patterns emerged revealing regional and inter-basin differentiation following a DDR for all scales considered. We further investigated present day environmental drivers and main evolutionary forces shaping the composition of prokaryotic communities populating the seafloor. In addition to the longitudinal structuration of communities, we confirm a systematic vertical stratification also reported at different depth scales in previous studies (Durbin and Teske, 2011; Orcutt et al., 2011; Jochum et al., 2017; Petro et al., 2019; Lloyd et al., 2020). We took advantage of the longitudinal extent of our dataset to investigate what processes might be at play in the assembly of microbial communities in and just below the bioturbation zone.

### On the Importance of Water Depth: An Environmental and Biogeographic Boundary?

The transition between the upper and lower continental slope, around 800–1,000 mbsl, is often associated with sharp local changes in sea bottom and water column conditions. It delineates the upper bathyal zone, found below the mesopelagic waters between 200 and ∼1,000 mbsl, and the lower bathyal zone (1,000–3,500/4,000 mbsl) (Watling et al., 2013; Costello et al., 2017). Our results indicate that this transition is also associated with marked changes in benthic microbial community structure as shown in the multidimensional analysis (Figure 4B). In terms of alpha-diversity, no decline with horizon depth was observed in the upper bathyal zone, while all samples of the lower bathyal zone exhibited such a trend (Supplementary Figure 2B). In terms of beta-diversity, communities present above 800 mbsl and below 1,200 mbsl clustered independently of temperature, which was very variable in this study, from the warmer Mediterranean to the colder Atlantic waters. This segregation was reflected in the larger amount of ASVs shared among shallow sites of the Mediterranean Sea and the Gulf of Cádiz, rather than with geographically closer but deeper sites (data not shown). Several non-mutually exclusive hypotheses could account for such sharp ecological transition, among which (i) the transition from piezotolerant to piezophilic microorganisms around 10 MPa, i.e., 1,000 mbsl (Fang et al., 2010; Cario et al., 2019; Scoma, 2020), and (ii) the nature of organic matter (OM) and its lability. Indeed, even though OM quantity did not vary significantly between depth zones (Supplementary Figure 5), more available OM may characterize sites closer to the shoreline in the upper bathyal zone (Seiter et al., 2005; Kallmeyer et al., 2012; Giovannelli et al., 2013), and.

When excluding the very distinct upper bathyal samples from the analysis, oceanic basin emerged as the second parameter influencing community structure: below 1,200 mbsl, communities segregated according to basin origin (Atlantic vs. Mediterranean). However, basins and temperature co-vary between the Atlantic and the Mediterranean, their respective contribution to beta-diversity has thus been difficult to disentangle, as illustrated by ordinations in Figures 4A,C. Other available environmental variables partially describing habitats (sediment granulometry, water content, OM) showed similarities between oceanographic basins (Figure 1B), and minimally contributed to the general beta-diversity variation partitioning analysis (5.2%, Supplementary Figure 4C). Finally, a latitudinal effect has been shown in other studies (Friedline et al., 2012). Here, no such correlation emerged, possibly due to the relatively narrow sampling zone, constrained between 20 and 40°N.

### Beyond Depth: Do Historical or Contemporary Parameters Drive Community Structure?

Given that the ecological processes influencing patterns of microbial community assembly are at play at any given time, it is necessary to consider their effects from a temporal as well as spatial point of view, thus distinguishing between historical and contemporary processes. In an influential review, Martiny et al. (2006) laid out a structured framework to interpret biogeographic data and the respective contribution of both types of processes. The authors defined “microbial habitats” as environments where microbial communities are structured by current ecological niche (defined by a set of biotic and abiotic parameters), while “microbial provinces” refer to regions that have undergone different historical processes, the legacies of which are visible in the contemporary structure of microbial communities. In the latter case, communities in equivalent niches but different provinces may harbor diverging communities. In our study, we thus used this framework and compared community and environmental similarity matrices to identify the contributions of these processes at different geographic scales.

At regional scale, we observed an important correlation of community similarity with both environmental similarity and geographic distance (Figure 2A), indicating the presence of both distinct microbial habitats and different microbial provinces. At distances beyond regional scale, however, only the DDR remained visible, while correlations with environmental similarity were largely lost (Figure 2B). Here, we cannot rule out the possibility that measurement of additional environmental parameters could lead to an increased link between community and environmental similarity, especially since the contribution of environmental selection to community structure was visible in the clustering patterns correlated with depth and temperature in the ordinations (Figures 4B,C). It has also been put forward that dormancy and the presence of microbial spores, abundant in marine sediments (Wörmer et al., 2019), can affect the biogeographic patterns observed at the microbial level (Locey et al., 2020). Nevertheless, in spite of this potential “noise” in our data, DDRs remained clearly apparent at both the inter- and intra-regional scales. Overall, our results show that the influence of historical processes such as dispersal limitation and past environmental conditions supersedes contemporary influences at inter-regional scale.

Previous studies (Martiny et al., 2006, 2011; Lecours et al., 2015) have highlighted differences in beta-diversity patterns depending on the scale considered. At local scale (Alborán Sea), both the DDR (Figure 5A) and the link between community and environmental similarity (Figure 5B) were apparent. Around 30% of ASVs were shared among all Alborán sites, quantitatively representing between 80 and 86% of reads. When focusing on the quantitative variation of these shared ASVs, the correlation between community and environmental similarity weakened (data not shown). This may reveal an influence of environmental selection mostly visible in the less abundant ASVs specific to each site, and/or dispersal limitation, with larger populations dispersing more easily and making up a core community of shared ASVs (Li et al., 2021). The presence of this biogeographic pattern at a scale of less than 10 km underlines the limited dispersal capability of benthic microorganisms (Zinger et al., 2011, 2014; Bienhold et al., 2016).

### Environmental Filtering and Ecological Drift in Subsurface Community Assembly

The important link between sediment horizon and community richness and composition was evident from the sample ordinations (Figure 4D), taxonomic composition (Supplementary Figure 2A), alpha-diversity patterns (Supplementary Figure 2B), and PERMANOVA analysis (*F* = 5.11, *p* = 0.001). The clearest changes in relative abundance were observed for Acidobacteria, Chloroflexi, and Bathyarchaeia, which all became more abundant deeper below the seafloor (Bienhold et al., 2016; Hoshino et al., 2020; Jørgensen et al., 2020; Lloyd et al., 2020; Vuillemin et al., 2020). In contrast, Desulfobacterota showed first an increase in relative abundance, before decreasing in deeper horizons of the upper bathyal zone, a pattern also described by Lloyd et al., 2020.

Recently, Petro et al. (2017, 2019) invoked selection of subsurface microorganisms locally from the surface community during burial as an important process driving subsurface community assembly. Here, we tried to quantify the relative contribution of stochastic vs. environmental processes at local scale using site-specific biomarker analysis. When comparing three adjacent sites (<10 km apart), we found that site-specific ASVs only marginally contributed to the total community (<1.8%, Figure 5D), whereas horizon-specific biomarkers, considered here as proxies for environmental filtering, were predominant (36.9–45.6% of reads). This finding is in line with the hypothesis of a strong environmental influence raised in previous work (Petro et al., 2017, 2019; Starnawski et al., 2017; Kirkpatrick et al., 2019; Marshall et al., 2019; Lloyd et al., 2020), even within the first decimeters of sediment. The observations from Petro et al. (2019) on the depth of influence of the bioturbation zone are also in line with the detection of macrofauna in a parallel metabarcoding study on these Mediterranean and Atlantic samples.

In addition, we observed increasing rates of distance-decay with increasing depth in the sediment throughout the entire transect (Figure 3 and Supplementary Table 3). In theory, an increase in DDR slope steepness can generally be explained by two processes: selection or lack of dispersal resulting in ecological drift (Hanson et al., 2012). In the case of selection, spatial auto-correlation of environmental parameters (e.g., salinity or temperature gradients) can lead to an increase of beta-diversity with distance. In this study, it is safe to assume that all environmental parameters possibly correlated with geographic distance (temperature, water depth) apply a similar pressure throughout the entire sediment horizons considered. We should thus observe parallel DDR regressions for the different sediment horizons, as community turnover rate would not vary with sediment depth. This is indeed what we observed at local scale (Figure 5A), suggesting that although the composition of surface communities differed, they changed simultaneously while being buried, most probably due the similar set of environmental conditions encountered. In contrast, we observed a clear increase of the distance-decay rate with sediment depth over the whole dataset (Figure 3 and Supplementary Table 3). We thus argue that, in this case, decreasing dispersal toward lower sediment horizons, leading to increasing ecological drift, is most probably responsible for the increase in microbial community differences across deep-sea sites with increasing sediment depth.

## Conclusion

This study presents a large scale, high definition characterization of the spatial distribution of benthic bacteria and archaea at the transition between two oceanic basins. Overall, we observed strong biogeographic patterns over the transition between the Mediterranean Sea and the Atlantic Ocean that depended on the scale considered. While at local and regional scale, community composition seemed to reflect both the influence of historical processes and of current environmental conditions, at the inter-regional scale the legacy of historical processes appeared more prevalent. Water depth, ocean basin, and water temperature were important environmental drivers of community structure. We found that in addition to environmental filtering, dispersal limitation and ecological drift emerged as influential processes in shaping the evolution of benthic microbial community composition with increasing depth in the sediment.

In the future, the importance of stochastic biogeographic processes in the assembly of early subsurface microbial communities could be further investigated by applying neutral and null-model approaches (Stegen et al., 2013; LaBrie et al., 2021), which might be more adapted to detecting the influence of drift in particular. In addition, part of the unexplained variation detected in our data is probably linked to biotic interactions with organisms not covered in this study (Gralka et al., 2020; Tobias-Hünefeldt et al., 2021), and may thus be further elucidated with metabarcoding data being generated for metazoans and protists in the scope of this project.

## Data Availability Statement

The dataset generated for this study has been submitted to the European Nucleotide Archive (ENA) under the following project: PRJEB33873. Details of the sample correspondence are provided in Supplementary Table 5. Additionally, the full dataset, including raw sequences, processed reads, and ASV tables, as well as bioinformatic scripts are available at: https://loimai.github.io/ABYSS_16S/.

## Author Contributions

LM and SA-H designed the study. SA-H, MB, JP, and CB carried out field and laboratory work. CO supplied ship time to conduct the sampling in Alborán Sea, Gulf of Cádiz, Ormonde seamount, and Formigas seamount. BT performed the bioinformatic and statistical analyses. BT, J-CA, LM, and SA-H contributed to data interpretation. BT, LM, and SA-H drafted the manuscript. All authors contributed to the final manuscript.

## Conflict of Interest

The authors declare that the research was conducted in the absence of any commercial or financial relationships that could be construed as a potential conflict of interest.

## Publisher’s Note

All claims expressed in this article are solely those of the authors and do not necessarily represent those of their affiliated organizations, or those of the publisher, the editors and the reviewers. Any product that may be evaluated in this article, or claim that may be made by its manufacturer, is not guaranteed or endorsed by the publisher.

## Footnotes

1
https://gitlab.ifremer.fr/abyss-project/abyss-pipeline

2
https://benjjneb.github.io/dada2/bigdata.html
