# Supplementary material for: Diversity and Biogeography of Bathyal and Abyssal Seafloor Bacteria and Archaea Along a Mediterranean—Atlantic Gradient
Source: Front Microbiol. 2021 Nov 1;12:702016. doi: 10.3389/fmicb.2021.702016 (PMC8591283; doi:10.3389/fmicb.2021.702016)
Supplement: Supplementary file 1 [file Data_Sheet_1.zip › Supplementary Data and Table S3-4 _Figure S1-4.docx]

Supplementary Material

# Supplementary Data

## PCR amplification

Prokaryotic barcodes were generated using the 515F-Y (5′- GTGYCAGCMGCCGCGGTAA-3′) and 926R (5′- CCGYCAATTYMTTTRAGTTT-3′) primers (Parada et al., 2016), and the Phusion High Fidelity PCR Master Mix with GC buffer (ThermoFisher Scientific, Waltham, MA, USA). PCR mixtures (25 μL final volume) contained 2.5 ng or less of DNA template with 0.4 μM concentration of each primer, 3% of DMSO, and 1X Phusion Master Mix. PCR amplifications (98°C for 30 s; 25 cycles of 10 s at 98°C, 30 s at 53°C, 30 s at 72°C; and 72°C for 10 min) of all samples were carried out in triplicate in order to smooth the intra-sample variance while obtaining sufficient amounts of amplicons for Illumina sequencing.

PCR triplicates were pooled and cleaned up using 1X AMPure XP beads (Beckman Coulter, Brea, CA, USA). Aliquots of purified amplicons were then run on an Agilent Bioanalyzer using the DNA High Sensitivity LabChip kit (Agilent Technologies, Santa Clara, CA, USA) to check their lengths and quantified with a Qubit fluorometer (Invitrogen, Carlsbad, CA, USA).

## Sequencing

### Amplicon library preparation

One hundred ng of amplicons were directly end-repaired, A-tailed at the 3’end, and ligated to Illumina compatible adaptors using the NEBNext DNA Modules Products (New England Biolabs, MA, USA) and NextFlex DNA barcodes (Bioo Scientific Corporation) with a liquid handler. This was done on a Biomek FX Laboratory Automation Workstation (Beckmann Coulter Genomics), able to perform up to 96 reactions in parallel. After two consecutive 1x AMPure XP clean ups, the ligated product were amplified using Kapa Hifi HotStart NGS library Amplification kit (Kapa Biosystems, Wilmington, MA), followed by 1x AMPure XP purification.

### Sequencing library quality control

Libraries were quantified by Quant-iT dsDNA HS assay kits using a Fluoroskan Ascent microplate fluorometer (ThermoFisher Scientific, Waltham, MA, USA) and then by qPCR with the KAPA Library Quantification Kit for Illumina Libraries (Kapa Biosystems, Wilmington, MA, USA) on a MxPro instrument (Agilent Technologies, Santa Clara, CA, USA). Library profiles were assessed using a high-throughput microfluidic capillary electrophoresis system (LabChip GX, Perkin Elmer, Waltham, MA, USA).

### Sequencing procedures

Metabarcoding libraries were characterized by low diversity sequences at the beginning of the reads, partly due to the presence of the primer sequence used to amplify tags. Low-diversity libraries can interfere in correct cluster identification, resulting in a drastic loss of data output. Therefore, loading concentrations of the metabarcoding libraries were normalised to 8–9 pM (instead of 12–14 pM for standard libraries) and contained a 20% PhiX DNA spike-in (instead of 1%) in order to minimize the impacts on the run quality. Libraries were sequenced on HiSeq2500 instruments (Illumina, San Diego, CA, USA) in a 250 bp paired-end mode.

## Sediment characterization

### Granulometric distribution

To determine granulometry of the sediments, the samples were processed using a Malvern Mastersizer 3000 and Hydro LV (Malvern Panalytical Ltd, Malvern, UK). For each sample, a spatula tip of matter was added to water and submitted to ultrasound treatment for 30 seconds at 100% of their power to break aggregates. The sample was then agitated for 30 seconds without ultrasounds in order to stabilise it. Following this, at least 4 granulometric measurements were performed in water, under a 2000 rpm agitation, with the following parameters: Mie theory with a refractive index of 1.52 and absorption of 0.1, and an obscuration rate between 0.5 and 15%. The result kept was the most repeatable value obtained. Between each sample, the machine underwent an automatic cleaning process.

### Humidity level and loss on ignition at 550°C

Approximately 2g of sediments were placed into dry and clean crucibles that had been previously weighed. The filled crucibles were then weighed before and after being placed overnight in a 100°C oven. Finally they were placed in a 550°C oven for 4 hours and weighed once more. Based on the weights measured, humidity level and loss on ignition values were computed.

# Supplementary Figures and Tables

## Supplementary Tables

**Supplementary Table S1**: Metadata table for all samples in the dataset, used to produce the figures included in the paper following the bioinformatic scripts available on github.

**Supplementary Table S2**: Number of reads by sample at every step of the bioinformatic processing workflow.

**Supplementary Table S3**: Values of linear regression parameters for log(Bray-Curtis similarity) = f(geographic distance) computed for each sediment horizon on the whole dataset.

| Horizon | Slope | Intercept | R^2^ | p-value |
| --- | --- | --- | --- | --- |
| 0 - 1 cmbsf | 0.000446 | -1.49 | 0.34 | < 2.2e-16 |
| 1 - 3 cmbsf | 0.000527 | -1.68 | 0.4 | < 2.2e-16 |
| 3 - 5 cmbsf | 0.000499 | -2.07 | 0.24 | < 2.2e-16 |
| 5 - 10 cmbsf | 0.000657 | -2.03 | 0.29 | < 2.2e-16 |
| 10 - 15 cmbsf | 0.000811 | -2.43 | 0.17 | 2.805e-13 |
| 15 - 30 cmbsf | 0.00255 | -0.680 | 0.97 | < 2.2e-16 |

**Supplementary Table S4**: Values of linear regression parameters for log(Bray-Curtis similarity) = f(geographic distance) computed for the Alborán Sea samples for each sediment horizon.

| Horizon | Slope | Intercept | R^2^ | p-value |
| --- | --- | --- | --- | --- |
| 0 - 1 cmbsf | -0.030 | -0.411 | 0.58 | 4.162e-08 |
| 1 - 3 cmbsf | -0.034 | -0.453 | 0.46 | 3.536e-06 |
| 3 - 5 cmbsf | -0.027 | -0.382 | 0.76 | 2.963e-12 |
| 5 - 10 cmbsf | -0.032 | -0.370 | 0.91 | < 2.2e-16 |
| 10 - 15 cmbsf | -0.027 | -0.451 | 0.64 | 3.189e-09 |
| 15 - 30 cmbsf | -0.022 | -0.541 | 0.37 | 4.68e-05 |
| 30+ cmbsf | -0.039 | -0.475 | 0.42 | 6.546e-08 |

**Supplementary Table S5:** Table of correspondence between sample names and ENA accession information.

## Supplementary Figures


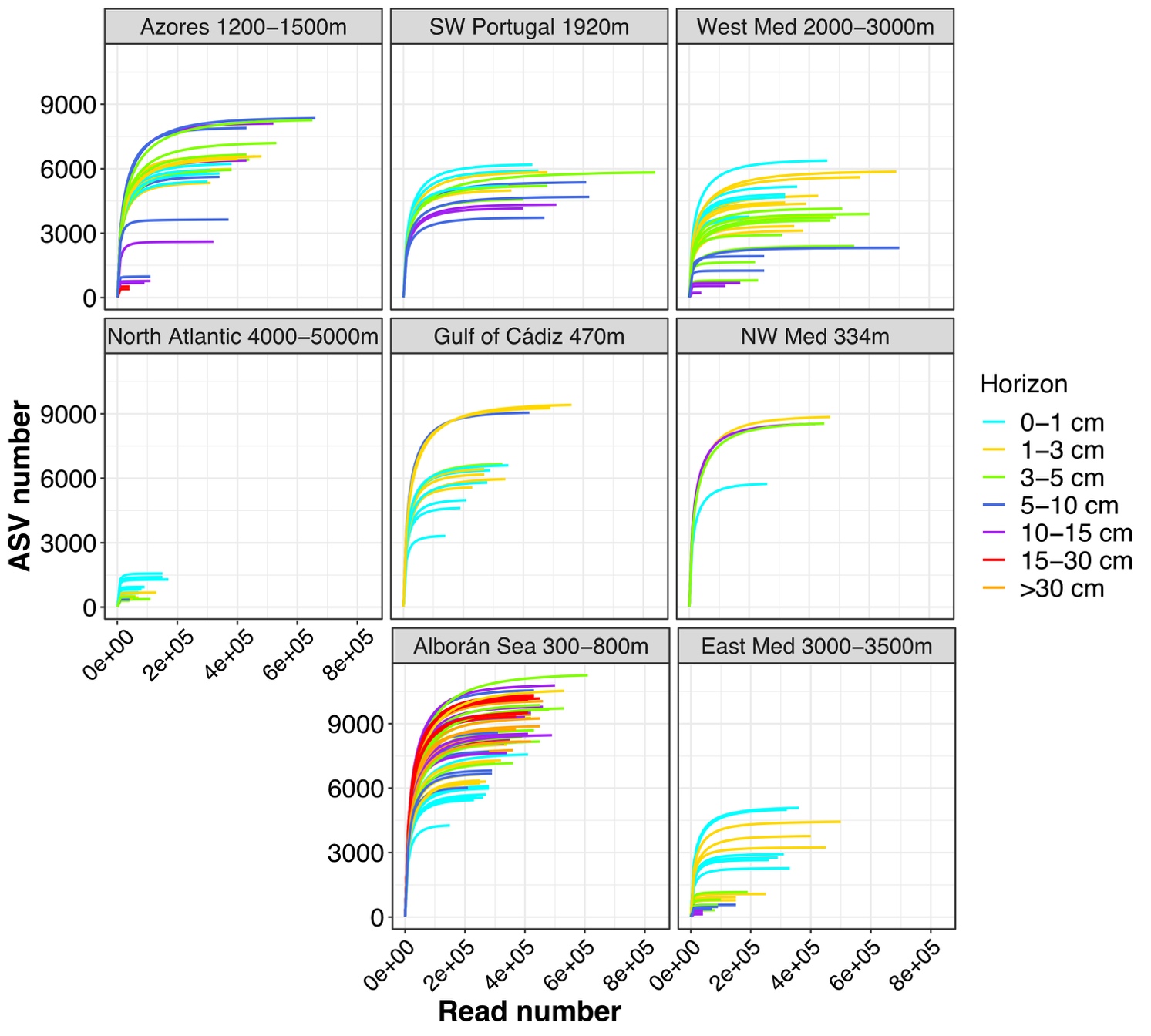


**Supplementary Figure S1**: Rarefaction curves for each sample in the metabarcoding dataset, arranged by sampling location and colored by sediment horizon.


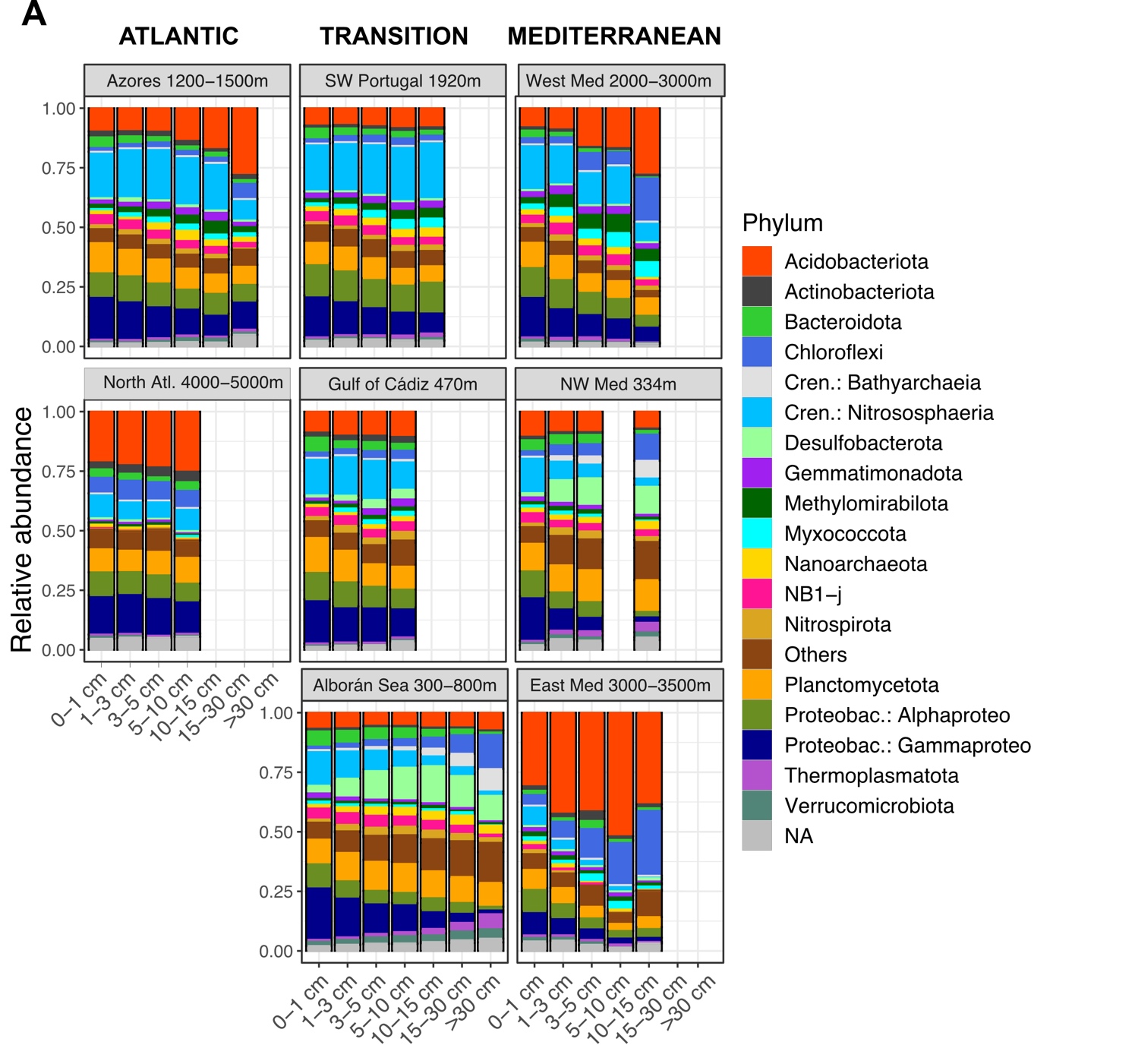


**
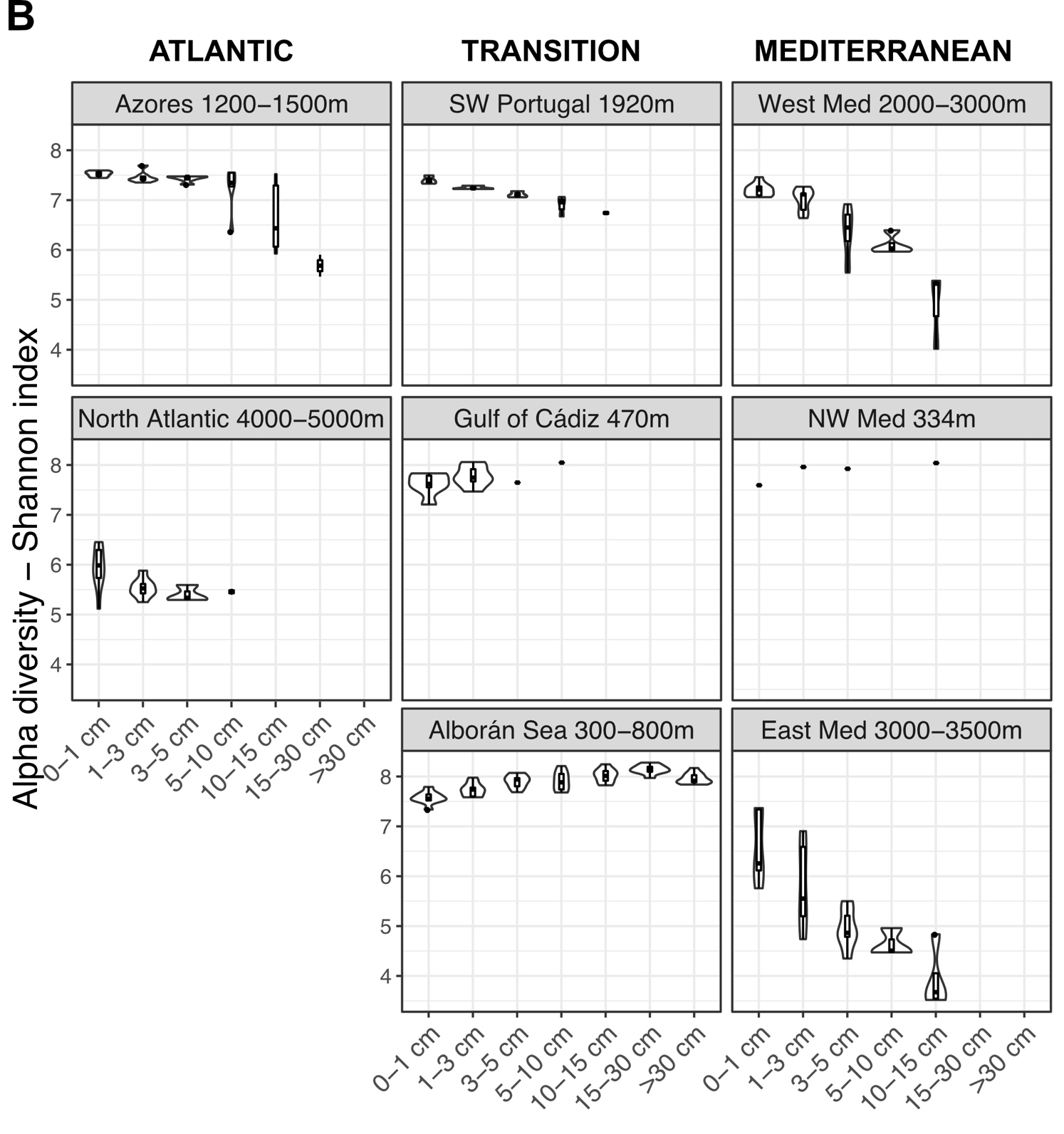
**

**Supplementary Figure S2**: **(A)** Relative abundance profiles of the 16 most abundant phyla in the dataset grouped by sampling location with increasing horizon depth (depth below the seafloor). Please note that Proteobacteria and Crenarchaeota members are identified at the class level for clarity. **(B)** Estimated alpha diversity (Shannon index) in samples grouped by sampling location and ordered by sediment horizon.


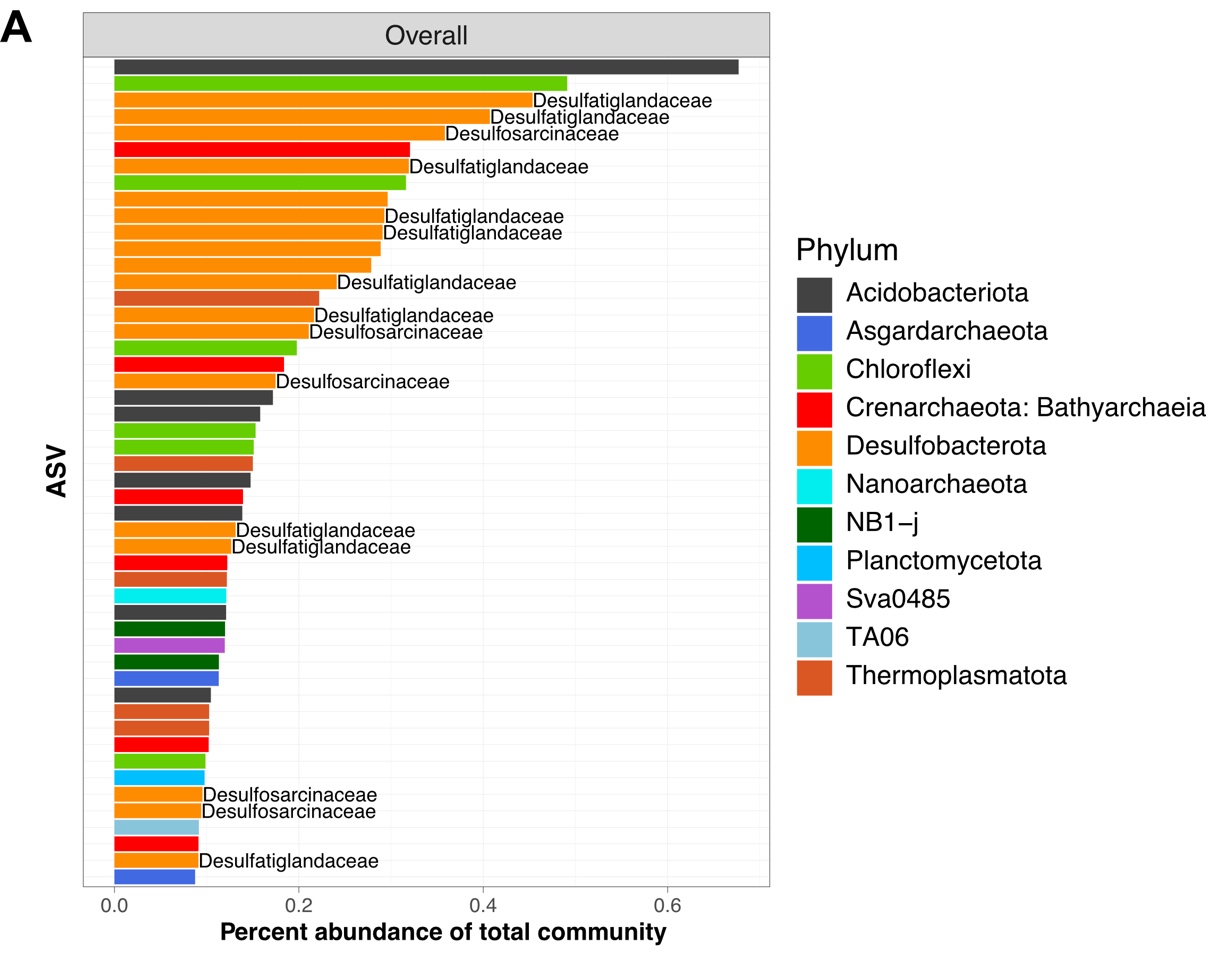
**
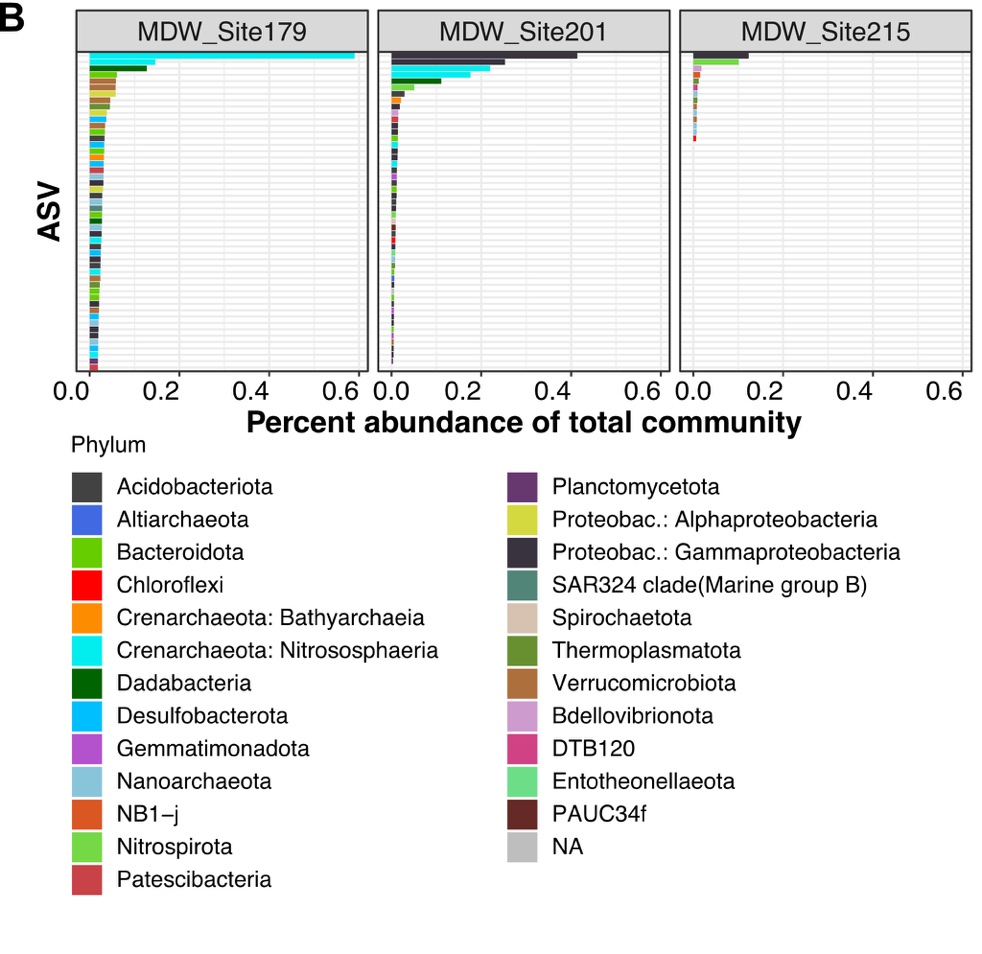
**

**Supplementary Figure S3**: Taxonomy of the 50 most abundant Alborán Sea biomarker ASVs for **(A)** the overall subsurface biomarker set and **(B)** the site-specific biomarker sets.


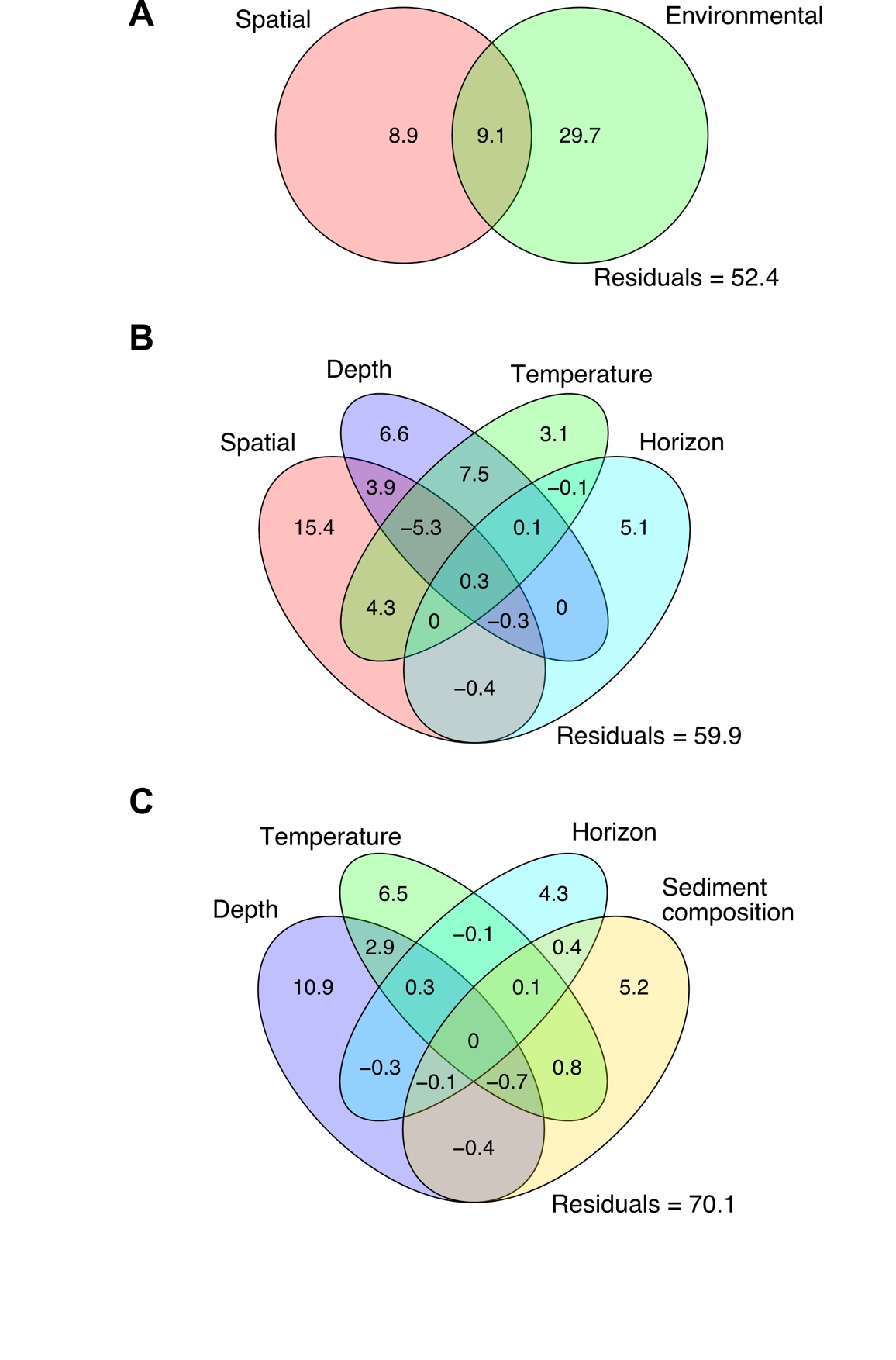


**Supplementary Figure S4**: Variation partitioning analysis of the data using Bray-Curtis dissimilarity. Partitioning according to **(A**) spatial vs environmental components, **(B**) spatial component, water depth, temperature, and sediment horizon, **(C**) water depth, temperature, sediment horizon, and sediment composition. The spatial component (**A, B**) refers to a combination of latitude, longitude and squared latitude. Sediment composition (**C**) refers to the combination of humidity level, organic matter content, mean granulometry and particle size (heterogeneity). Finally, the environmental component in (**A**) is calculated using all the sediment variables mentioned above, combined with water depth, temperature, distance from shore and horizon depth. Fractions are annotated with the obtained adjusted R square for each explanatory variable or matrix, for which significance was tested. Values for the intersections are found by subtracting different models and underline the possible redundancy of the explanatory variables. They cannot be assigned significance, and negative values are a possible artefact of the analysis.


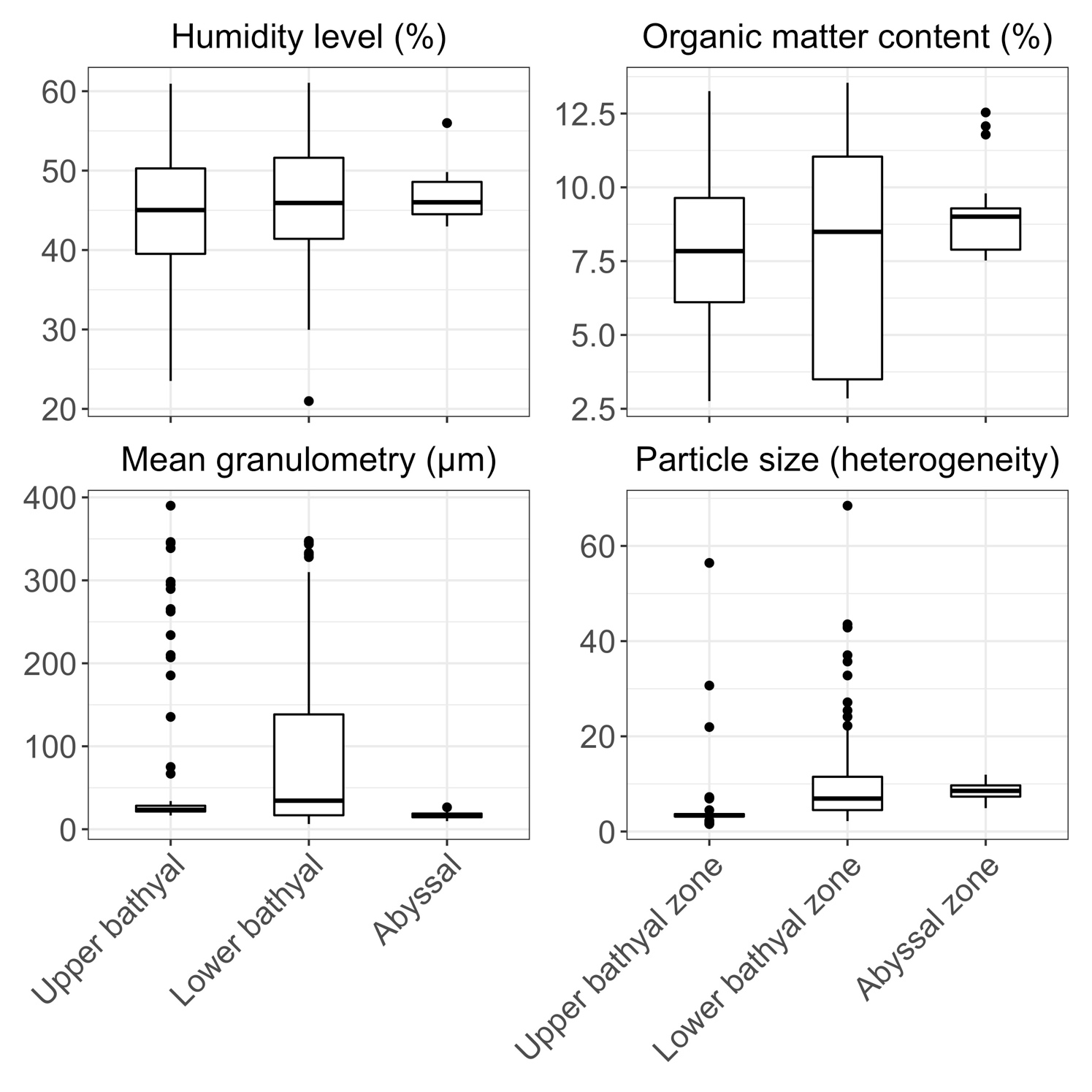


**Supplementary Figure S5**: Evolution of sediment characteristics in the three depth zones targeted by the longitudinal sampling scheme. Only mean heterogeneity of particle sizes significantly differed between depth zones.
